# Dr. Harmon J. Ashley

**Published:** 1908-07

**Authors:** 


Dr. Harmon J. Ashley, of Machias, N. Y., died at his home in that village June 5, 1908. He graduated at the University of Buffalo, medical department, in 1875, and became one of the prominent physicians in Cattaraugus County. He was 58 years old at the time of his death.

